# Investigating the needs of family caregivers of older stroke patients: a longitudinal study in Iran

**DOI:** 10.1186/s12877-020-01670-0

**Published:** 2020-08-28

**Authors:** Mansoureh Ashghali Farahani, Shiva Bahloli, Roohangiz JamshidiOrak, Fatemeh Ghaffari

**Affiliations:** 1 Nursing Care Research Center (NCRC), School of Nursing and Midwifery, Iran University of Medical Sciences, Rashid Yasemi St, Valiasr Ave, Tehran, Iran; 2 School of Nursing and Midwifery, Iran University of Medical Sciences, Tehran, Iran; 3 Department of Statistics & Mathematics, School of Health Management and Information Sciences, Iran University of Medical Sciences, Rashid-Yasemi Street, Vali’asr Avenue, Tehran, Iran; 4 Nursing Care Research Center, Health Research Institute, Babol University of Medical Sciences, Shahid Motahari St, Ramsar, Babol, Mazandaran ZIP Code 4691714141 Iran

**Keywords:** Family caregivers, Need, Older adults, Stroke, Longitudinal studies

## Abstract

**Background:**

The high burden of care associated with older stroke patients is a factor that threatens the health of family caregivers. Identifying the needs of family caregivers in this group of patients can help provide effective solutions. The present study aimed to determine the needs of family caregivers of older stroke patients.

**Methods:**

The sample size of this longitudinal study included 200 family caregivers of older stroke patients from two hospitals in Iran. Data collection included demographics, responses to family caregivers’ needs questionnaires, and the Barthel Index which was taken in four stages including admission time, pre-discharge, two weeks and 12 weeks post-discharge.

**Results:**

The results showed that all participants at all stages of the study identified “respect for the patient when providing education, treatment, or rehabilitation” as one of their needs. There was a statistically significant relationship between the older adult survivor’s age and the number of family caregivers’ needs two weeks post-discharge (*p* = 0.012) and 12 weeks post-discharge (*p* = 0.008). There was a significant relationship between the patient’s hospitalization period and the number of caregivers’ needs three months after the patient’s discharge (*p* = 0.028), and a significant statistical relationship between the pre-discharge physiotherapy of the patients and the number of their caregivers’ needs during the two weeks post-discharge (*p* = 0.018). There was also a statistically significant relationship between the patient’s level of dependence and the number of caregivers’ needs (*p* = 0.0001). On the contrary, there was no significant relationship between the sex, place of living, and underlying disease history of the patient and the number of caregivers’ needs (*p* > 0.05).

**Conclusion:**

The results of the present research indicate that the total number of caregivers’ needs decreases with increasing duration of the disease. However, respite and care provision planning by other family members, seeking assistance from professional caregivers, and the search for community support resources can help reduce the burden of care of caregivers and give them the opportunity to meet their needs in different dimensions of patient care provided.

## Background

According to the World Health Organization (WHO) in 2013, the number of people aged 65 and over had surpassed children under the age of 5 for the first time in the world. The elderly population is projected to reach 2 billion worldwide in 2050 [1]. According a 2016 census in Iran, the elderly population was reported to be about 9.3% of the total population [2]. Older adults experience many chronic diseases, including diabetes, hypertension, and stroke during their life [3]; stroke being one of the most common which results in a patient’s physical, mental, and social disability [4]. Stroke is the leading cerebrovascular disorder in the world and the third leading cause of death worldwide after cardiovascular diseases and cancer [5]. In Western countries, it occurs in 1–3 people per 1000 populations per year [4], and in Iran, it occurs in 372 individuals per 100,000 populations [6].

Advanced age is one of the risk factors for stroke. Reduced cognitive ability and disability in performing daily activities of life caused by age exacerbate the symptoms and complications of the disease, and imposes a widespread burden of care on the family and healthcare systems. Furthermore, numerous studies show that despite the efforts of the family and the medical professionals, the outcomes of rehabilitation programs are not satisfactory [7, 8]. Researchers have attributed the causes of failure of treatment and rehabilitation programs to the long-term dependence of older adults on healthcare services and the high cost of these services, lack of follow-up treatment due to fatigue and burnout of family caregivers, and lack of access to family support systems in the society [9].

Considering the need for long-term care and widespread consequences, stroke greatly affects the lives of older adults and their families who care for and support them [10]. Following a stroke, the lives of stroke survivors and their families change drastically that it becomes difficult to return to their lives pre-stroke, which is why it is regarded as a family disease. The consequences of strokes and the rehabilitation process depend largely on the role of the family and its support [11]. The main function of family is to meet the individual needs of family members [12]; however, due to their unpreparedness and sudden requirement to provide extensive care for an older patient, they have a tendency to neglect their own needs as well as the needs of other family members [10].

Family caregivers perform multiple duties and need to learn new roles, such as using home-based care equipment, drug management, and measures related to the prevention of recurrent strokes. They often face problems such as anxiety, confusion, job loss, and many physical problems due to the burden of care [12, 13]. The co-occurrence of old age and stroke increases the burden of care of family caregivers, especially in those caring for older patients suffering from chronic diseases such as diabetes, hypertension, dementia, and Alzheimer’s. Under such conditions, the severity and burden of care reaches a point where it can lead to depression among family caregivers [14].

In most countries, both formal and informal systems are responsible for meeting the care needs of the older adults. Official surveillance systems include professional and healthcare systems, and formal public and private systems. Informal care is also provided by family members, friends, and relatives. In an elderly care system, these two parts complement each other [15]. In most countries, healthcare providers and community health policymakers are trying to adopt strategies to prevent re-admission of older patients due to care needs for chronic diseases in treatment centers and high expenditure due to disease complications. Therefore, the role of family caregivers as the patient’s companions at the time of admission and assigning the responsibility of patient care to them following discharge are important strategies applied in most communities [16]. In Iran, due to the lack of government centers for rehabilitation, post-discharge care for the older stroke patients, and excessive payment for care in private centers and home care services, it is very common for families to take on the main responsibility of providing care to patients [5]. However, social systems provide insufficient support to these older patients and their family caregivers.

Although family and patient education is one of the important accreditation indicators in clinical centers and is considered a right in Iran, in practice, older patients and their family caregivers are deprived from specialized training [17, 18]. This has caused family caregivers to encounter different health problems in various aspects of elderly care in Iran. This group of people carries an extreme burden of care due to the lack of awareness on how to provide care, how to get help, or because of false cultural beliefs that cause them to reject any help offered by other family members or relatives.

Unfortunately, the first coping strategy adopted by family caregivers is to neglect their needs in different dimensions, which can threaten both their health and the outcome of the care provided [19]. Considering the burden of care and the stress that family caregivers experience while providing care to older patients, the maintenance and continuity of family care needs to be identified and family caregivers’ needs fulfilled [20]. Previous studies indicate the different needs of family caregivers in different stages of the disease, and if such needs are not identified and effective interventions are not implemented, caregivers’ health status is threatened, which in turn can lead to a decline in the life expectancy and quality of life index of this group of people [21–23]. However, most relevant studies focus only on the needs of family caregivers at certain time periods. The changing needs of family caregivers from admission to discharge and post-discharge periods are not addressed [24, 25]. Furthermore, a review of literature showed that studies in Iran focused on the needs of patients and there was no study on the needs of family caregivers. Therefore, the present research aimed to determine the needs of family caregivers of older stroke patients in Iran at different stages of their disease.

## Methods

### Study design

This research is a longitudinal study conducted in two hospitals in Urmia County in West Azerbaijan Province of Iran. These treatment centers were selected since older stroke patients are referred to them from all counties of the province.

### Study population

The researcher contacted the two hospitals and prepared a list of older stroke patients who were diagnosed and admitted to the neurology ward. Following this, a list of the main family caregivers who were present in hospitals as companions was prepared. In this study, the inclusion criteria was that participants must be present at the hospital as the older patient’s companion at the time of admission, aged over 18 years old, assumed the main responsibility of providing care to the older stroke patient at home, and had the ability to read and write in Persian. Exclusion criteria included: the death of the patient during the study, the assignment of responsibility of care to a formal caregiver at home, and the assignment of responsibility of care to another person in the family during the study period. Individuals who met the inclusion criteria from the list of family caregivers were randomly selected to participate in the study. The sample size included 210 people, with *P* = 0.8, d = 0.054, and a 95% confidence interval.

### Data collection

The first and second stages (admission time and pre-discharge): In the first stage, the demographic questionnaire were given to the patients and their caregivers. The questionnaire consisted of two main parts—patient and family caregivers’ characteristics, followed by the family caregivers’ needs questionnaire, which was distributed only among the caregivers. In the second stage, in addition to the family caregivers’ needs questionnaire, the Barthel index was also completed by the family caregivers. For the third and fourth stages (two and 12 weeks post- discharge), the researcher coordinated home visits with the participants via telephone calls at each stage, and again provided the participants with the family caregivers’ needs questionnaire and the Barthel index.

### Measurements

#### Two-part demographic characteristics questionnaire

- *Part one:* This part contained questions about the demographic characteristics of the older patient, including age, gender, place of residence, underlying diseases, and information related to the disease, such as length of stay and pre-discharge physiotherapy. The data required in this part was obtained from the patient’s medical records.
- *Part two:* This part included questions about the family caregivers’ demographic characteristics, including age, gender, level of education, occupation, marital status, religion, their relationship with the patient, and living conditions (living with or without the patient).

#### Needs questionnaire for family caregivers of patients with stroke

The questionnaire was based on the needs questionnaire for family caregivers by Marutz and Kreutzer and includes 43 items and six dimensions, including items on health information (13 items), instrumental support (9 items), emotional support (8 items), patient care participation (5 items), professional support (4 items), and community networks (4 items). The presence and absence of a need is assigned a score of 1 and 0, respectively. The total scores of each dimension comprise the scores for that dimension, and the overall score is calculated by summarizing the scores obtained for each dimension [12]. In the present study, after obtaining permission from the original author of the questionnaire and completing the translation and re-translation process, content validity of the tool was determined. For this purpose, the final re-translated version along with the original questionnaire were presented to 10 faculty members of the Faculty of Nursing and Midwifery, Iran University of Medical Sciences, who were experts in the field of strokes to assess its validity. Following the necessary corrections, these questionnaires were used. The reliability of the questionnaires was evaluated and obtained a Kuder–Richardson 20 score of 0.95 among 30 family caregivers of older stroke patients [26]. The data of the pilot study were not used in the final analysis. The questionnaire measures the needs of the family caregivers of patients with stroke is available from the developers at file:///C:/Users/nasim/Downloads/ppa-9-449.pdf.

### Barthel index

This questionnaire measures the level of an individual’s dependence on others while performing his/her daily activities. This questionnaire includes questions about intestinal and bladder control, personal hygiene, toilet use, eating, moving from bed to chair and vice versa, walking, dressing up, using stairs, and bathing. The scores assigned to different parts of this index range from 0 to 15 and the total possible score ranges from 0 to 100. Gaining scores of 0–24, 25–49, 50–74, 75–90, 90–99 and 100 from this index indicate complete, strong, moderate, mild, very low, and lack of dependence, respectively [27]. The reliability and validity of the questionnaire has been investigated. The validity of the questionnaire on the needs of family caregivers of stroke patients has been evaluated by nine experts. The content validity index (CVI) was calculated to be 0.99. The internal consistency of the questionnaire was also reported to have a Kuder–Richardson 20 score of 0.89–0.93% for the three stages of need assessment [26]. The Barthel index was not considered at the first stage of the study while the patient’s situation was unstable during their hospitalization in internal neurology wards. In the pilot study, 30 family caregivers of older stroke patients who were randomly selected from the study environment were provided with a questionnaire. After collecting the questionnaires, the reliability of the questionnaire was calculated and obtained a score of 0.95 using Kuder–Richardson 20. It should be noted that the pilot study data was not used in the final analysis. The Barthel index is available from the developers at https://www.mdcalc.com/barthel-index-activities-daily-living-adl

### Statistical analysis

Statistical analysis was carried out using SPSS ver. 18. To verify the assumption of normality, Kolmogorov–Smirnov test was used. The data were analyzed using descriptive statistics (frequency, percentage, mean, and standard deviation) and inferential statistics (Spearman correlation coefficient with zero, Mann-Whitney, and Kruskal-Wallis). The study set the significance level at *p* < 0.05.

## Results

Four individuals were excluded due to death of the patient over the course of the study and six others due to the reassignment of responsibility of care to another family member. Data of these people was not analyzed. According to the Barthel index at the time of discharge, more than half of the patients (52%) had “complete” dependence on their caregivers. Over time, this number decreased; none of the patients had more than a moderate dependence on their caregivers 3 m post-discharge. “Moderate” dependence was observed among (61%) the patients two weeks post-discharge and in nearly half (46%) of them at three months post-discharge. Over time, the percentage of patients with mild dependency on their caregivers increased from 0 to 39% (Table 1).

**Table 1:** Frequency distribution of the level of elderly’s dependence on family caregivers according to the Barthel index in three different times

Dependence	Stage		
	Pre-discharge	2 weeks after discharge	12 weeks after discharge
	***N***(%)	***N***(%)	***N***(%)
Complete	104 (52)	32 (16)	0 (0)
Strong	62 (31)	44 (22)	30 (15)
Moderate	34 (17)	122 (61)	92 (46)
Mild	0 (0)	2 (1)	78 (39)
very low	0 (0)	0 (0)	0 (0)
lack of dependence	0 (0)	0 (0)	0 (0)
Total	200 (100)	200 (100)	200 (100)

The results showed that in the health information dimension, all caregivers required “respect for the patient when providing education, treatment, or rehabilitation” at all times. Another need identified by almost all of the caregivers was “honest answers to their questions” during all instances. With regard to the community networks dimension, almost all caregivers required “understanding of the patient’s problems by other family members” at the time of admission and discharge. Furthermore, all four needs under the professional dimension which were brought up at the time of admission and discharge were considered very important. “Having information on how to manage the patient’s emergency conditions” was identified as a need by nearly all caregivers at a later stage (two weeks and three months post-discharge). With regard to the lateral support dimension, results showed that more than 93% of the caregivers required “having enough rest or sleep” at all times. As for the patient care participation dimension, 100% of the caregivers expressed the need to “help the patient adapt to their physical care so that they feel more comfortable while doing activities, such as cleaning, bathing, and massaging” at the time of admission and discharge; but over time, fewer caregivers felt such a need (Table 2).

**Table 2:** Frequency of family caregivers’ care needs in four stages of study in different dimensions

Needs	Stage of study			
	Admission time	Pre-discharge	2 weeks after discharge	12 weeks after discharge
	%	%	%	%
**Health information**				
Respect the patient when performing education, treatment or rehabilitation	100	100	100	100
Providing information on disease progress	98	98	98	88
To have my question answered honestly	100	100	100	99
To have information on medication and treatment	99	99	99	99
To have information on the patient’s physical problems	98	98	98	98
To have information on the patient’s rehabilitation progress	96	97	93	70
To be assured that the best possible medical care is being given to the patient	99	98	82	55
To be informed about all changes in the patient’s medical status, such as blood	100	100	88	62
To be informed about the exams and laboratory results daily	99	99	7	4
To have explanations from professionals given in terms I can understand	97	98	88	64
To have information on the patient’s thought problems	93	92	29	14
To know how to communicate with patients	97	97	83	61
To have information about dying and hospice care when the patient disease reaches the end	87	87	32	25
**Community support network**				
Conveying advice with the help of professionals	92	92	68	41
To have other family members understand the patient’s problems	98	98	95	86
To have support from family and friends	96	96	94	87
To discuss my feelings about caring for the patient with other friends or family	92	92	85	75
**Professional support**				
Being informed of how to handle the medical emergencies of the patient	99	99	100	99
To have comprehensive information for the patient eg, rehabilitation programmes, physical therapy	94	94	88	70
Being informed on what to do when the patient became upset or acted strange	100	99	84	65
To discuss with heath care workers on how much the patient can do independently	99	99	81	58
**Instrumental support**				
To have help from other members of the family in taking care of the patient	99	99	95	87
To get enough rest or sleep	99	100	99	93
To have information about financial assistance, eg, physical and mental disability benefits, medical expenses, catastrophic illness benefits, hospital care in seriously ill low-income subsidy	98	98	83	48
To get a break from my problems and responsibilities	95	95	92	87
To have time to spend with friends	52	46	17	11
To have information about homecare (eg, home rehabilitation, day care, respite care)	98	99	97	77
To have information about assistive devices (eg, beds, wheelchairs, oxygen, suction machine, ventilator)	94	93	42	24
To have information on patients’ long-term care (eg, nursing home, respiratory care center)	81	81	39	29
To have help keeping the house (eg, shopping, cleaning, cooking, etc.)	92	92	91	89
**Emotional support**				
Help remaining hopeful about the future	100	100	92	76
To be encouraged to ask others to help out	96	96	93	88
To express my feelings about the patient with someone who has gone through the same experience	96	96	69	50
To have my partner or friends understand how difficult it is for me	96	96	91	74
Help in getting over my doubts and fears about the future	97	96	75	65
To have time to go to temple or church	88	89	88	77
To be reassured it is usual to have strong negative feelings about the patient	96	95	68	57
To discuss my feelings about the patient with a professional, eg, anxious, worry, sad, feeling guilty, ange	95	95	83	61
**Involvement with patient care**				
To learn how to help patients stand up, shift and rehabilitation techniques	98	98	92	76
To help patients deal with physical care to make patients more comfortable, eg, grooming, bathing and massage	100	100	91	63
To learn how to prepare meals for patients	98	98	73	29
To learn nasogastric tube and urinary catheter care	86	83	20	12
To learn patients’ wound care	91	90	35	22

The results show that in the first and second stages of the study, the highest average scores of needs were in the professional support and health information dimensions. Similar to the admission and discharge time periods, needs under the “professional support” dimension had the highest average score among the six dimensions two and 12 weeks post-discharge (Table 3).

**Table 3:** Average needs of family caregivers in different dimension in different stages of study

Domain	Stage of study							
	Admission time		pre-discharge		2 weeks after discharge		12 weeks after discharge	
	Mean	SD	Mean	SD	Mean	SD	Mean	SD
**Health information**	97.2	3.63	97.2	3.69	76.7	31.9	63.9	32.53
**Community networks**	94.5	3	94.5	3	85.5	12.5	72.3	21.53
**Professional support**	98	2.71	97.8	2.5	88.3	8.34	73	18.02
**Instrumental support**	89.8	15.25	89.2	17.23	72.8	31.17	60/6	32.55
**Emotional support**	95.5	3.38	95.4	3.02	82.4	10.36	68.5	12.43
**Involvement with patient care**	94.6	5.9	93.8	7.16	62.2	33	40.4	27.63

The results show that the mean age of participating caregivers was 42.8 ± 11.79 years. The majority of caregivers (70%) were women. More than a third of the women (38%) had elementary education and most of them (60%) were housewives. There was no statistically significant relationship between the needs of family caregivers and demographic variables (Table 4).

**Table 4:** Relationship between demographic characteristics of family caregivers and their needs at four different times

Variable		No		Stage				Statistical Test
				Admission time	pre-discharge	2 weeks after discharge	12 weeks after discharge	
Age		200	Correlation Coefficient	0.093	0.091	0.121	0.090	Spearman ‘s correlation coefficient
			*P*_value	0.355	0.37	0.23	0.373	
sex	Female	140	Mean	49.91	49.85	52.11	52.89	Mann–Whitney
	Male	60	Statistic value	51.88	52.02	46.75	42.92	
				0.32-	0.351-	0.85-	1.259-	
			P_value	0.748	0.725	0.396	0.308	
Education	Elementary	76	Mean of ranks	58.04	57.47	47.79	44.58	Kruskal–Wallis
	High school	44	Statistic value Statistic value	49.95	51.02	53.70	53.48	
	Diploma	52		34.72	45.17	48.69	55.75	
	Bachelor and above	28		41.61	40.64	56.18	52.14	
				5.189	3.951	1.247	2.72	
			*P*_value	0.158	0.175	0.742	0.436	
Job	Employee	80	Mean of ranks	44.96	44.64	42.55	41.77	Mann–Whitney
	House wife	120	Statistic value	44.28	44.23	45.41	45.78	
				−0.119	−0.037	−0.49	−0.687	
			*P*_value	0.905	0.971	0.624	0.492	
Relatives	Daughter	130	Mean of ranks	48.24	37.78	35.67	37.46	Kruskal–Wallis
	Son	50		35.4	35.68	36.04	34.94	
	husband	20		46.3	37.35	42.45	42.90	
			Statistic value	0.298	0.159	1.447	1.138	
			*P*_value	0.862	0.934	0.485	0.157	
Residence with the elderly	Yes	84	Mean of ranks	48.5	48.54	35.57	54.44	Mann–Whitney
	No	116		51.85	51.92	48.28	47.65	
			Statistic value	−0.603	- o.592	−0.904	−1.159	
			*P*_value	0.547	0.554	0.466	0.247	
Marital status	Single	32	Mean of ranks	45.62	45.78	49.22	48.03	Mann–Whitney
	Married	166		50.84	50.81	50.15	50.38	
			Statistic value	−0.684	−0.659	−0.119	− 0.3	
			*P*_value	0.494	0.51	0.905	0.764	

The mean age of older patients was 70.8 ± 7.25 years. The number of older women was more than older men and most of them (73%) lived in counties surrounding the city of Urmia. More than half of the older patients (54%) were admitted to the hospital for one week or less, and most of them (85%) had not undergone physiotherapy prior to discharge. There was a statistically significant relationship between demographic variables related to the older patients, namely age, length of stay, and physiotherapy and the number of caregivers’ needs at certain time periods studied. On the other hand, there was no statistically significant relationship between the patient’s gender, place of residence, and underlying diseases with the number of caregivers’ needs at different time period studied (*p* > 0.05) (Table 5).

**Table 5:** Relationship between demographic characteristics of the older adult and the their caregivers’ needs in four different times

Variable		N		Stage				Statistical Test
				Admission time	pre-discharge	2 weeks after discharge	12 weeks after discharge	
**Age**		200	Correlation Coefficient	0.115	0.121	0.251	0.263	Spearman’s correlation coefficient
			P_value	0.257	0.232	0.012	0.008	
**sex**	Female	118	Mean of ranks	53.14	52.45	53.98	53.64	Mann–Whitney
	Male	82		46.71	47.7	45.49	45.98	
			Statistic value	- 1.119	−0.828	−1.446	−1. 303	
			P_value	0.264	0.408	0.148	0.193	
**City**	Urmia	146	Mean of ranks	49.09	49.21	50.22	52.27	Mann–Whitney
	Other cities Suburbs	54		54.31	53.69	51.26	45.70	
			Statistic value	−0.821	−0.681	−0.160	- 1.008	
			P_value	0.411	0.493	0.873	0.314	
**length of stay**	One week or less	148	Mean of ranks	52.97	51.80	46.21	44.65	Mann–Whitney
	More than a week	52		47.60	48.98	55.53	57.37	
			Statistic value	0.948	−0.497	−1.607	−2.191	
			P_value	0.443	0.619	0.108	0.028	
**Physiotherapy**	Yes	30	Mean of ranks	59.60	60.43	66.77	55.10	Mann–Whitney
	No	170		49.42	48.85	47.63	49.68	
			Statistic value	−0.907	−1.477	^*^- 2.365	−0.677	
			*P*_value	0.364	0.140	0.018	0.498	
**Underlying diseases**	Yes	160	Mean of ranks	49.66	49.42	51.21	51.18	Mann–Whitney
	No	40	Statistic value	53.85	54.83	47.68	47.78	
				−0.593	−0.765	−0.489	−0.471	
			*P*_value	0.553	0.444	0.625	0.638	
**Dependence**		200	Correlation Coefficient	–	−0.057	^*^-.335	0.378	Spearman’s correlation coefficient
			*P*_value		−0.573	0.001	0.0001	

Furthermore, there was a statistically significant relationship between the level of the patient’s dependence, as measured by the Barthel index at discharge, two weeks post-discharge, three months post-discharge, within two weeks post-discharge, and twelve weeks post-discharge, with the number of caregivers’ needs. The Spearman’s correlation coefficient of the relationship between these two variables indicated that the higher the level of the patient’s dependence was, the higher the number of caregivers’ needs would be (Table 5).

## Discussion

Exploring the needs of family caregivers who take care of older stroke patients is particularly important. The study assessed the needs of family caregivers of older stroke patients across four stages. Needs under the dimensions of “professional support” and “health information” scored the highest in the first and second stages of the study. Consistent in all stages of the study, the need which scored highest under the professional support dimension was “having information about how to deal with an older patient when he or she exhibits discomfort or abnormal behavior.”

The highest needs under the health information dimension included “respecting the older patient during training, treatment, or rehabilitation,” “the need to answer my questions honestly,” and “knowledge of changes in the older patient’s medical condition, such as blood pressure and heart rate.” Tsai et al. (2015) found that the average scores for health information dimension were higher than those in other dimensions [12]. In their studies on common problems of family caregivers, Ossee et al. (2006) and Deeken et al. (2003) also referred to the caregivers’ lack of information and specialized knowledge about the disease as the most important problems and needs [28, 29].

Dorsey et al. (1998) identified the following needs of caregivers: “to be included in discharge plan,” “to have questions answered honestly,” “to understand how to communicate better with the stroke patient,” and “to know how procedures and planning are done” [30] . Hayashi et al. (2013) have also proposed five main needs or concerns of caregivers who take care of stroke patients hospitalized at acute care units, including “stroke prevention,” “information on drugs,” “recovery time,” “availability of hometown hospitals,” and “ability of stroke survivor to conduct activities of daily living.” The same study also indicated that having information on how to maintain the patient’s health condition or how to prevent further strokes were among the main needs of 83.7% of the caregivers [21] .

The results of this study prove that professional support and receiving health information during hospitalization are among the common needs of caregivers. This may be due to the severe conditions which occur as a consequence of stroke in older adults (such as restlessness, fear and panic, changes in sleep pattern and nutrition, as well as behaviors such as aggression and rejection), which cause the family caregivers to experience shock, uncertainty, and confusion regarding the treatment process as they witness the unfavorable cognitive and physical conditions of the patient right after the stroke [31].

Caregivers also deal with physical problems such as fatigue, sleep alteration, exhaustion, and pain, as well as experience emotions including worry, anxiety, and uncertainty. The change in their social role may also result in their social isolation [32]. They discover that being a caregiver becomes an inseparable part of their lives and an inevitable duty. Fear of the unknown in such a situation causes the need for support and reception of information from health care providers [33].

Health professionals ought to provide and perform rehabilitation programs based on the older patient and his or her caregiver’s needs during the patient’s hospitalization to provide both the patient and caregiver with solutions for dealing with the consequences of a stroke and to establish their roles [34]. De Oliveira et al. (2011) placed emphasis on the readiness of health care providers for providing needs-based education and an appropriate educational atmosphere for caregivers. For this purpose, the hospitalization period is the best time to conduct a collaborative and regular educational program [35]. This is the best opportunity for nurses to reduce the caregivers’ stress level by establishing effective communication with them and providing essential information about the disease process, its signs and symptoms, and how to manage it.

The results of this study showed that in the third stage of the study (two weeks post-discharge), among the six dimensions studied, needs under the “professional support” and “community networks” dimensions scored the highest. Furthermore, in most stages of the study, the need for “family members to understand the problems of the older patient” was considered more important than other needs in most stages of the study.

While the caregivers receive professional support from the medical professionals (especially nurses at the time of admission), as soon as the patient arrives home, the caregiver faces many problems which often stem from their lack of awareness and lack of support from the community and other members of the family. Due to the chronic nature of stroke and extensive need for care of the patient shortly after discharge, this disease causes caregivers to face physical, mental, and psychological fatigue, and if they are married or employed, many problems concerning their family and job also surface [36].

Factors such as the distance of the caregiver’s residence from the patient’s house, the older patient’s need for a long-term care, lack of experience for caring for an older patient, and lack of knowledge about the needs of an older stroke patient are among the main reasons why other family members do not participate in providing care. In these circumstances, most of the caring duties are delegated to the member who took care of the older patient before the stroke [37]. However, measures such as making other family members understand the situation and having them assist and share the care duties, establishing effective communication with the treatment team or community health nurses, and receiving psychological counseling services can help family caregivers to not feel lonely and presents an opportunity for them to meet their own or their family’s needs [38].

These findings are consistent with the results of studies by Tsai et al. (2015) and Mohammadi et al. (2011) where family caregivers believed that they needed time to deal with their personal affairs to be able to fulfill other social and personal roles besides providing care to the patient. An effective solution may be to engage other family members in helping the main caregiver by increasing their level of commitment and responsibility [39]. Older patients and family caregivers tend to receive support from the treatment staff and are followed up after discharge. Unfortunately, older stroke patients are not socially supported by any organization or association following their discharge from the hospital in Iran. Many of these patients require important services to meet their needs for urination (catheterization) and defecation, nutrition, speech, prevention of bed sores, physiotherapy, etc., and the heavy burden of these responsibilities is assumed by their caregivers [40]. Caregivers need support and consultation from close friends and relatives as well as the treatment team to cope with problems with care, reduce tension, and increase their capabilities through spirituality [41, 42].

Based on the results, older stroke patients’ needs 12 weeks after discharge were significantly less as compared to two weeks post-discharge.

Furthermore, needs under the dimensions of “professional support” and “community networks” scored highest, whereas needs related to the “participation in older patient care” dimension scored lowest in the fourth stage of the study (12 weeks post-discharge). With regard to the professional support dimension, the most important needs included “having information on how to manage the older patient’s emergency conditions.” The most important need under the community networks support dimension was “getting support from family members and friends.”

These findings are consistent with the results of the study carried out by Tsai et al. (2015). Caregivers may face emergency situations while taking care of an older stroke patient, or the patient may undergo a stroke during recovery. Failure to manage such circumstances can be very stressful and be associated with serious consequences such as the death of the patient [43].

In addition to the stressful lives of caregivers, due to the patient’s old age, the recovery process is very slow and complications of the disease along with other problems associated with aging can increase the burden of care for caregivers. However, the provision of information to caregivers by physicians or nurses during follow-up visits, encouraging caregivers to accept other family members’ assistance, and training family members to encourage them to support the patient and caregiver can improve the quality of care and maintain the health status of caregivers in various dimensions [44]. Some studies have focused on home care services and respite care to reduce the care burden of caregivers [45, 46].

Moreover, the study found that needs of family caregivers in the pre-discharge period were lower than those during the admission time. The number of family caregivers’ needs was also lower in the post-discharge time than in the pre-discharge period. These results are probably due to the fact that the longer the duration of care was, the more caregivers become familiarized with the care needs of their patients. Regular visits and rehabilitation post-discharge can also be beneficial in dealing with disease progression and rehabilitation, which can lead to a gradual reduction in their needs. Furthermore, the results of the present research suggest that there is no significant relationship between the number of family caregivers’ needs at the studied time periods with the demographic variables of family caregivers. These findings are similar to the results of studies carried out by Tsai et al. (2015) and Hayashi et al. (2013).

The results of statistical tests showed a statistically significant relationship between the older patients’ age and the number of caregivers’ needs at two weeks post-discharge and 12 weeks post-discharge. In other words, the number of caregivers’ needs increased in relation to the age of their older patient. This relationship can be explained such that older patients usually have a low-level self-care ability, resulting in increasing the responsibilities of the caregiver and more needs. On the other hand, fear of death because of age, understanding the complex and severe conditions, and the diminished self-worth due to the reduction of abilities are among the main reasons for older patients becoming dependent on caregivers. This issue is exacerbated by the older patient’s unwillingness to perform self-care activities and requesting for more of the caregivers’ service. This results in an increase in the number of caregivers’ needs especially in the support aspect [47].

The present study also found a relationship between the length of stay and the number of caregivers’ needs within 12 weeks post-discharge. Tsai et al. (2015) also found a relationship between the length of stay in the hospital and the number of caregivers’ needs at all four stages [12]. This may be due to the fact that the length of stay is related to the severity of a patient’s disease and that the more complex condition of their disease results in family caregivers having more needs. The financial burden imposed on the family by long-term hospitalization, the need for long and frequent referrals to the hospital for visiting the patient or participating in care, as well as the increased chance of older patient to acquire infections at the hospital are among the other reasons for the increased number of caregivers’ needs in relation to an increased period of hospitalization [48].

The results showed a statistically significant relationship between pre-discharge physiotherapy and the number of caregivers’ needs two weeks post-discharge. This may be due to the fact that older patients who underwent physiotherapy usually felt more physically dependent on their caregivers, which resulted in caregivers having more needs than others. Physiotherapy results in less dependence of the older patient and reduces the burden of care [21], however, older patients who are on the younger side, or those who deserve more caring needs, are usually placed on the waiting list due to the lack of facilities in Iranian hospitals or other limitations. Therefore, families don’t understand the outcomes of physiotherapy during hospitalization. Unfortunately, physiotherapy centers which provide home services are usually private institutes which are not supported by insurance. As a result, families are restricted to limited public service or tolerate the burden of care imposed by the older patient’s dependency [30].

Furthermore, the findings indicate a statistically significant relationship between the level of patient’s dependence and the number of caregivers’ needs. These findings are consistent with the results of the study carried out by Tsai et al. (2015). Since older stroke patients are dependent on others in carrying out many of their daily activities, such as eating, urinating, defecation, and bathing, and are dependent on caregivers for physiotherapy as well as changing their physical position, it is a tiresome and stressful experience to provide care for them.

There was also no significant relationship between gender and comorbid diseases with the number of their caregivers’ needs (*p* > 0.05), similar to findings in Tsai et al.’s study (2015). However, the results of a study by Chafjiri et al. (2017) indicate that the female caregivers have more needs, especially in providing physical support, as compared to male caregivers. This occurred because in most cases, the caregivers are the wives of the older patients and are already suffering from physical problems associated with their age, resulting in a more complex situation. Most duties for elderly care are delegated to women in Iranian culture. They face caring challenges at different stages of treatment, whether during hospitalization or post-discharge, and they have to play role of caregiver while carrying out their duties as wives, mothers, or employees [41]. They become more of a caregiver than a wife or a daughter in such cases [49].

Furthermore, there was no relationship found between the number of needs and underlying diseases in this study. However, Hesamzadeh et al. (2016) showed that underlying diseases such as diabetes, coronary artery diseases, etc., result in more pressure for care, increases caregiver fatigue, and increases caregivers’ needs especially for information, support, and the patient’s collaboration in self-care due to the chronic nature of such diseases and the need for long-term care [18] .

There was also no significant relationship between the place of residence of older patients with the number of their caregivers’ needs (*p* > 0.05). This finding is consistent with the results of Tsai et al. (2015). Most of the caregivers in this study were not living at the same place as the patients they provided cared to. This gave them the opportunity to assign care to other family members and allowed them more time to rest and deal with other daily activities, which can contribute to decreasing the number of caregiver’s needs at different stages of the disease [41].

## Conclusion

Professional support, health information, and community networks support were the main dimensions of caregivers’ needs at all four time periods. The findings also indicate that most needs of family caregivers arose in the first and second stages of the study. Understanding the needs of caregivers of older stroke patients by the treatment team, holding family-centered training sessions during admission time, educating caregivers on how to care for the older patients and how to meet their needs during the post-discharge stage and rehabilitation, as well as providing training on the older patient’s conditions, drugs and treatment, and disease progression can help decrease needs of the caregivers.

Severity of the stroke was not explored as a confounding factor in physiotherapy sessions and the number of caregivers’ needs at any stage of this research. This should be explored further in future studies.

### Limitations

- The cognitive and emotional states of the family caregivers while answering the questionnaires were one of the limitations of this research. In this regard, the researcher made an effort to provide a relaxed and stress-free environment for family caregivers to complete the questionnaires at a time when their older patients’ conditions were stable.
- The sample size of the present study was small, which limits the generalizability of results to other family caregivers of older stroke patients. Therefore, the findings of the study should be interpreted with caution.
- In this study, data collection was carried out using a self-reporting tool. The researchers made efforts to emphasize that the information contained in the questionnaires would be analyzed anonymously, and that the results would be presented in general so as to reduce bias in answering the questionnaires.

## Recommendations for future researches

- This study only focused on the needs of family caregivers of older stroke patients. Therefore, it is suggested that future studies be carried out considering the needs of family caregivers of older patients with other chronic diseases.
- Future research should consider studying the experiences of nurses and other members of the care team in relation to the needs of caregivers of older stroke patients and the barriers to meeting such needs will be effective in improving the system.
- The study was only conducted in two public hospitals. It is recommended to carry out future studies in clinics and private centers.

## Data Availability

Data generated or analysed during this study are included in this published article and are available from the corresponding author on reasonable request.

## Abbreviations

WHO: World Health Organization
CVI: Content validity index
